# Research on indoor positioning method based on LoRa-improved fingerprint localization algorithm

**DOI:** 10.1038/s41598-023-41250-x

**Published:** 2023-08-26

**Authors:** Honghong Chen, Jie Yang, Zhanjun Hao, Macidan Ga, Xinyu Han, Xiaotong Zhang, Zetong Chen

**Affiliations:** College of Computer Science and Engineering, Northwest Normal University, Gansu, China

**Keywords:** Computer science, Computational science

## Abstract

Traditional fingerprint localization algorithms need help with low localization accuracy, large data volumes, and device dependence. This paper proposes a LoRa-based improved fingerprint localization algorithm-particle swarm optimization-random forest-fingerprint localization for indoor localization. The first improvement step involves creating a new exceptional fingerprint value (referred to as RSSI-RANGE) by adding the Time of Flight ranging value (referred to as RANGE) to the Received Signal Strength Indication (RSSI) value and weighting them together. The second improvement step involves preprocessing the fingerprint data to eliminate gross errors using Gaussian and median filtering. After noise reduction, the particle swarm optimization algorithm is used to optimize the hyper parameters of the random forest algorithm, and the best RSSI-RANGE value is obtained using the random forest algorithm. The Kriging method is then used for interpolation to establish an offline fingerprint database, and the final online recognition and localization are performed. Experimental results demonstrate that the first improvement step improves localization accuracy by 53–57% in different experimental scenarios, while the second improves localization accuracy by 25–31%. When both steps are combined, the localization accuracy is improved by 58–63%. The effectiveness of this method is demonstrated through experiments.

## Introduction

With the continuous development and popularization of Internet of Things (IoT) technology, there is an increasing demand for Location Based Services (LBS) that provide accurate location information^1^. In indoor positioning, fingerprinting algorithms have become a research hotspot due to their low cost, portability, and the fact that they do not require the establishment of complex path loss propagation models. However, traditional fingerprinting algorithms have many limitations, such as limited accuracy, device dependence, and large data volume^2^.

LoRa is a low-power, wide-coverage, and indoor-penetration wireless communication technology^3^, which can be used in fingerprint algorithms. Firstly, LoRa technology has strong signal penetration capability, transmitting signals even in indoor environments. Secondly, due to the low-power characteristics of LoRa technology, the lifespan of nodes is extended, reducing the frequency of battery replacement and the overall operational cost of devices. Finally, the low cost of LoRa technology reduces the overall cost of devices. In addition, LoRa technology supports self-organizing networks, does not require wireless communication authorization, and can be used in large-scale scenarios.

**Figure 1:**
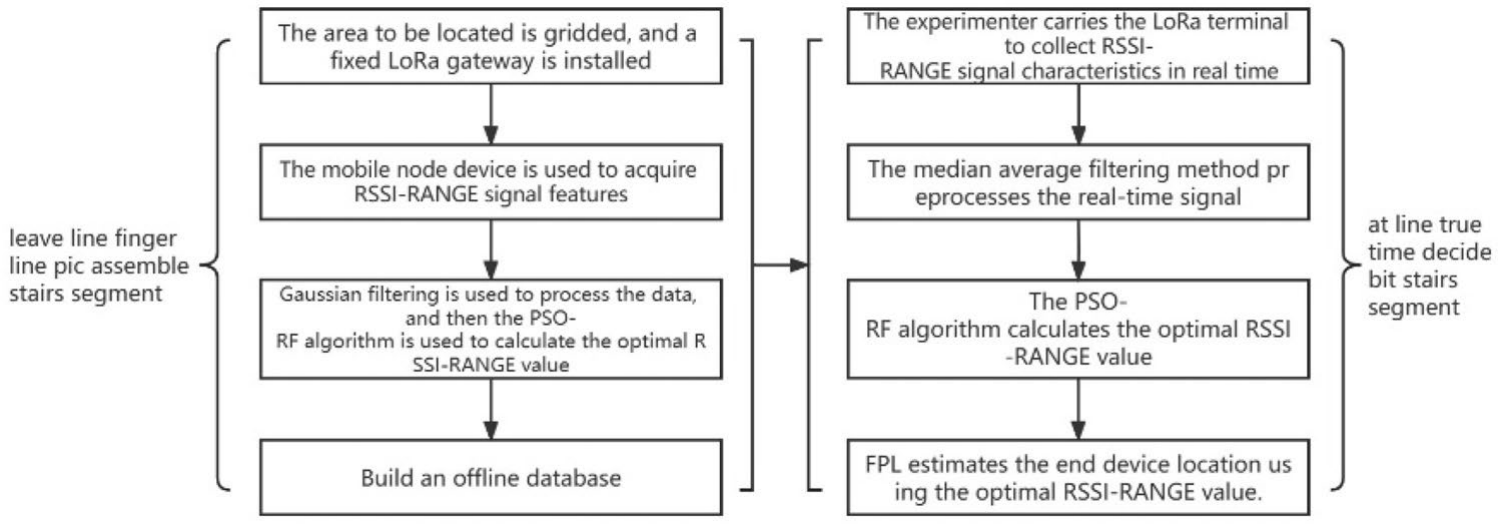
PSO-RF-FPL algorithm flowchart.

Based on this, this method proposes a LoRa-based improved fingerprint localization algorithm for indoor positioning named PSO-RF-FPL. Gaussian and median average filtering is used for fingerprint preprocessing during the database establishment and online positioning process to eliminate coarse fingerprint errors. Additionally, the RSSI values sampled by LoRa signals in indoor positioning are often affected by various factors, such as noise, scattering, signal reflection, and interference. This method proposes a new fingerprint feature, RSSI-RANGE, which is formed by weighting the indoor RSSI values with the Time of Flight (TOF) distance measurement to reduce the impact of these factors. Kriging interpolation is used to reduce the problem of large data volumes during fingerprint collection. Then, the PSO algorithm is used to optimize the hyperparameters of the RF algorithm, and the optimal fingerprint value is selected to establish an offline fingerprint database. Finally, the online recognition stage is completed for positioning and recognition. The flowchart of the algorithm is shown in Fig. 1. The contributions of this method are as follows:

1. The cost of indoor positioning using traditional fingerprint algorithms is reduced by utilizing the characteristics of LoRa. The proposed method improves the positioning accuracy and reduces the data volume by using the RSSI-RANGE value and Kriging interpolation. The experimental results demonstrate that the PSO-RF-FPL algorithm applies to various environments and effectively improves the positioning accuracy of traditional fingerprint algorithms.
2. The algorithm’s validity is demonstrated by using 3–4 gateway devices. This study provides a theoretical basis for further increasing the number of gateway devices to improve positioning accuracy and apply the algorithm to large-scale scenarios.

The method proposed in this method utilizes the characteristics of LoRa to reduce the cost of indoor positioning of traditional fingerprint algorithms. At the same time, RSSI-RANGE values and Kriging methods are used to improve positioning accuracy and reduce data volume.

## Related work

Indoor positioning technology relies on both positioning techniques and algorithms. Currently, there are two main positioning techniques: sensor-based and signal-based. Sensor-based techniques include sound sensors^4^, optical sensors^5^, and infrared sensors^6^, but they require the installation of numerous sensors and have low accuracy^7^. In contrast, signal-based techniques have become the focus of research due to their advantages in ease of installation and high accuracy^8^. Therefore, various wireless signals, such as Wi-Fi, ZigBee, RFID, BLE, UWB, LoRa, Sigfox, and NFC, are widely used in indoor positioning research.

There are several algorithms for indoor positioning, including proximity algorithms, triangulation algorithms, multipoint positioning algorithms, maximum likelihood algorithms, and fingerprint positioning algorithms. Fingerprint positioning algorithms are widely used in large-scale environments such as shopping centres, markets, and campus buildings because of their characteristics. Alhomayani et al.^9^ discussed the advantages and disadvantages of various fingerprint types used in indoor positioning and looked at future research directions. Cui et al.^10^ proposed a method to process fingerprint data based on skewness-kurtosis normality test and Kalman filter fusion, and the positioning accuracy was improved by 60% compared with the traditional Kalman filter method. Lian et al.^11^ proposed a KPCA-ELM joint positioning algorithm that uses the nonlinear properties of KPCA (kernel principal component analysis) to allow for the replacement and dimensionality reduction of original RSS data and construct new features. The article demonstrates that the algorithm can effectively reduce the impact of noise on RSSI values and improve accuracy by comparing different WIFI AP signal points. Marwan Alfakih et al.^12^ proposed a new fingerprint probability algorithm that can improve the accuracy of device positioning using Wi-Fi signal strength in indoor environments, with accuracy improvements ranging from 5.1 to 21.5% in different environments. However, this method is limited due to the relatively short distance of Wi-Fi signals in practical applications. Jait Purohit et al. proposed an interpolation-assisted fingerprint positioning system architecture^13^. They addressed the issue of large LoRa network size and wide coverage range by proposing a deep autoencoder method that effectively solves the problem of missing/outlier samples in the LoRa network. Suroso et al. applied interpolation techniques to reduce the time and effort required to collect fingerprint data. They compared the classic pattern-matching algorithm and the minimum Euclidean distance algorithm. The latter performed better in accuracy and precision, while the random forest algorithm performed better in reducing the maximum estimation error^14^.

In the study, factors such as the effectiveness of RSSI data processing and the stability of LoRa indoor propagation will affect the absolute positioning accuracy, so it is necessary to comprehensively analyze these factors, use appropriate hardware devices and antennas, and develop more effective RSSI data processing strategies to improve the accuracy of LoRa-based indoor positioning^15–18^. In related research fields, various methods have been proposed to solve similar problems, such as Kalman filters, moving average filters, multipath fading modelling, and calibration methods^19–25^.

## Proposed method

The fingerprinting localization (FPL) algorithm involves placing multiple wireless signal sources in a specific area. The surrounding environment affects the wireless signals emitted, forming a one-to-one correspondence between the received signal strength indication (RSSI) and the location.

FPL is based on Bayesian theory to estimate the probability of the terminal node being located in various areas. The deployment area is defined as ${\mathrm{X}}_{1}\sim {\mathrm{X}}_{\mathrm{n}}$, where ${\mathrm{X}}_{\mathrm{i}}\in \mathrm{X}$ and ${\mathrm{X}}_{\mathrm{i}}$ is defined as the coordinate $\left({\mathrm{x}}_{\mathrm{i}},{\mathrm{y}}_{\mathrm{i}}\right)$. The vector s represents the RSSI-RANGE values sampled by n gateways, $\mathrm{s}=\left\{\left({\mathrm{RSSI}-\mathrm{RANGE}}_{1}\right),\left({\mathrm{RSSI}-\mathrm{RANGE}}_{2}\right),\ldots ,\left({\mathrm{RSSI}-\mathrm{RANGE}}_{\mathrm{n}}\right)\right\}$. ${\mathrm{RSSI}-\mathrm{RANGE}}_{\mathrm{n}}$ denotes the RSSI-RANGE value sampled by the nth gateway. Each location ${\mathrm{X}}_{\mathrm{i}}$ has a corresponding vector s. During the online phase, the n gateways' RSSI-RANGE values form the vector ${\mathrm{s}}^{\mathrm{^{\prime}}}$, and the goal is to find the area with the highest probability, that is, to determine $\mathrm{argmaxp}\left({\mathrm{X}}_{\mathrm{i}}|{\mathrm{s}}^{\mathrm{^{\prime}}}\right)$. According to Bayes' formula, the posterior probability formula can be obtained.

1 $$ {\text{p}}\left( {{\text{X}}_{{\text{i}}} {\text{|s}}^{\prime } } \right) = \frac{{{\text{p}}\left( {{\text{s}}^{\prime } {\text{|X}}_{{\text{i}}} } \right){\text{p}}\left( {{\text{X}}_{{\text{i}}} } \right)}}{{{\text{p}}\left( {{\text{s}}^{\prime } } \right)}} $$

In the formula, $\mathrm{p}\left({\mathrm{X}}_{\mathrm{i}}\right)$ is the prior distribution of the location, $\mathrm{p}\left({\mathrm{s}}^{\mathrm{^{\prime}}}\right)$ is the signal strength distribution, independent of the location. Since a uniform prior distribution is set here, the denominator $\mathrm{p}\left({\mathrm{s}}^{\mathrm{^{\prime}}}\right)$ and prior distribution $\mathrm{p}\left({\mathrm{X}}_{\mathrm{i}}\right)$ can be ignored in Bayes' formula. Therefore, it is necessary to compare $\mathrm{p}\left({\mathrm{s}}^{\mathrm{^{\prime}}}|{\mathrm{X}}_{\mathrm{i}}\right)$. The maximum likelihood estimation method is used to estimate the location of the terminal device. The likelihood function (2) is $\mathrm{p}\left({\mathrm{s}}^{\mathrm{^{\prime}}}|{\mathrm{X}}_{\mathrm{i}}\right)$, and the location estimated by formula (3) is where the likelihood function is maximized.

2 $$ {\text{p}}\left( {{\text{s}}^{\prime } {\text{|X}}_{{\text{i}}} } \right) = \frac{{{\text{F}}\left( {{\text{s}}^{\prime } ,{\text{X}}_{{\text{i}}} } \right)}}{{\mathop \sum \nolimits_{{{\text{j}} = 1}}^{{\text{n}}} {\text{F}}\left( {{\text{s}}^{\prime } ,{\text{X}}_{{\text{i}}} } \right)}} $$

3 $$ {\text{X}}^{\prime } = {\text{X}}_{{\text{i}}}^{{{\text{argmaxp}}\left( {{\text{s}}^{\prime } {\text{|X}}_{{\text{i}}} } \right)}} $$

The likelihood function in Eq. (2) can ignore the denominator using maximum likelihood estimation. $\mathrm{F}\left({\mathrm{s}}^{\mathrm{^{\prime}}},{\mathrm{X}}_{\mathrm{i}}\right)$ in Eq. (4) is obtained by the sum of probabilities from all gateways.

4 $$ {\text{F}}\left( {{\text{s}}^{\prime } ,{\text{X}}_{{\text{i}}} } \right) = \sum\nolimits_{{{\text{j}} = 1}}^{{\text{k}}} {{\text{weight}}_{{{\text{gw}}_{{\text{j}}} }} \times {\text{p}}\left( {{\text{RSSI}}_{{\text{j}}}^{\prime } {\text{|gw}}_{{\text{j}}} ,{\text{X}}_{{\text{i}}} } \right)} $$

The conditional probability $\mathrm{p}\left({\mathrm{s}}^{\mathrm{^{\prime}}}|{\mathrm{X}}_{\mathrm{i}}\right)$ is the probability that the gateway ${\mathrm{gw}}_{\mathrm{j}}$ measures the RSSI-RANGE value at location ${\mathrm{X}}_{\mathrm{i}}$ which is obtained by comparing ${\mathrm{s}}^{\mathrm{^{\prime}}}$ with the data ins.

This method combines the Bayesian and trilateration algorithms to achieve more accurate localization results. The initial position is calculated using the trilateration algorithm, and then the Bayesian algorithm is used to refine the initial position. In the Bayesian algorithm, the prior and posterior probability is combined with the weighted distance calculated in the trilateration algorithm to obtain a more accurate position estimate. The following are the steps for combining the weighted distances:

1. Use the trilateration algorithm to calculate the initial position, assuming the calculated target position is (x_0,_ y_0_).
2. Use the Bayesian algorithm to refine the initial position. The Bayesian algorithm is a probability-based localization method using prior and posterior probability to calculate the target position. In indoor localization, signal strength and gateway location information can be used to calculate the prior and posterior probability. Assume that the target position calculated by the Bayesian algorithm is (x_1_, y_1_).
3. Weighted formulas can be based on either errors or signal strength. This method selects a weighted formula based on errors for combining the trilateration and Bayesian algorithms.

Assuming that the target position calculated by the i-th algorithm is (x_i_, y_i_)and the proper position is (x_t_, y_t_), the distance between them can be represented as ${\mathrm{d}}_{\mathrm{i}}=\sqrt{{\left({\mathrm{x}}_{\mathrm{i}}-{\mathrm{x}}_{\mathrm{t}}\right)}^{2}+{\left({\mathrm{y}}_{\mathrm{i}}-{\mathrm{y}}_{\mathrm{t}}\right)}^{2}}$. The reciprocal of ${\mathrm{d}}_{\mathrm{i}}$ is used as the weight, i.e., ${\mathrm{w}}_{\mathrm{i}}=\frac{1}{{\mathrm{d}}_{\mathrm{i}}}$. The final weighted average position can be calculated using the following formula:

5 $${\mathrm{x}}_{\mathrm{f}}=\frac{\sum {\mathrm{w}}_{\mathrm{i}}{\mathrm{x}}_{\mathrm{i}}}{{\mathrm{w}}_{\mathrm{i}}},{\mathrm{y}}_{\mathrm{f}}=\frac{\sum {\mathrm{w}}_{\mathrm{i}}{\mathrm{y}}_{\mathrm{i}}}{{\mathrm{w}}_{\mathrm{i}}}$$

Compute the weighted average of the positions obtained by each algorithm according to their weights, and obtain the final position coordinates (${\mathrm{x}}_{\mathrm{f}},{\mathrm{y}}_{\mathrm{f}}$).

**Figure 2:**
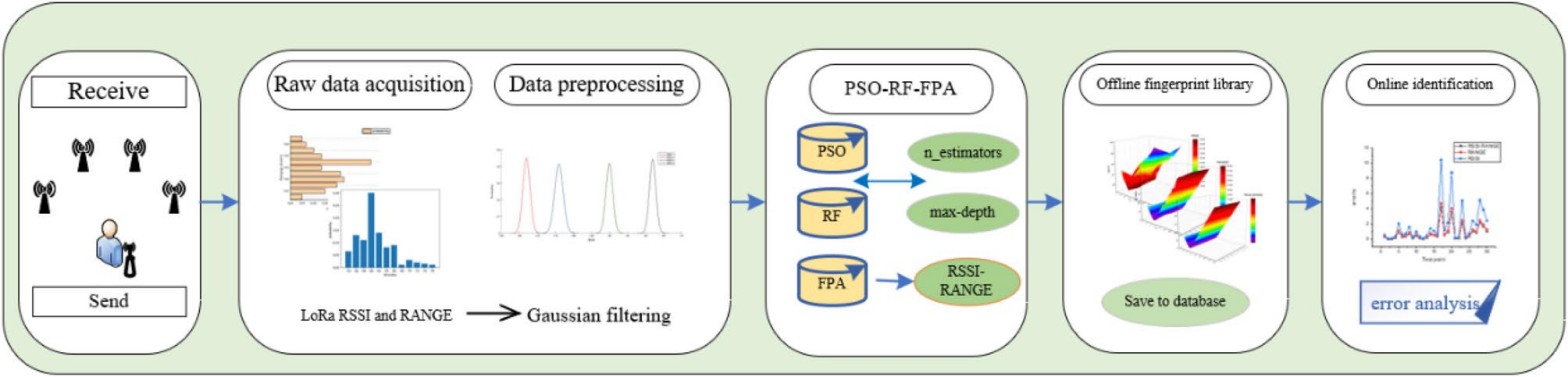
Workflow diagram.

Four steps are needed to perform indoor positioning using the proposed PSO-RF-FPL method: collecting RSSI and TOF measurements, data preprocessing, building an offline fingerprint database, and online positioning. The workflow diagram is shown in Fig. 2.

### Data collection and preprocessing

**Figure 3:**
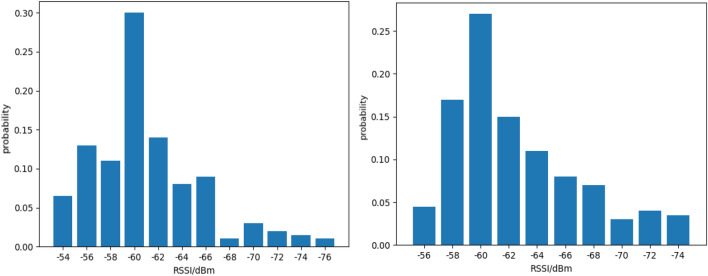
RSSI values at specific test points.

In indoor wireless signal propagation, the RSSI values collected from the same anchor at the exact location can vary continuously over time due to the fluctuation of wireless signals. Figure 3 shows 200 RSSI values collected using LoRa devices at several test points, with the antenna facing vertically upward.

**Figure 4:**
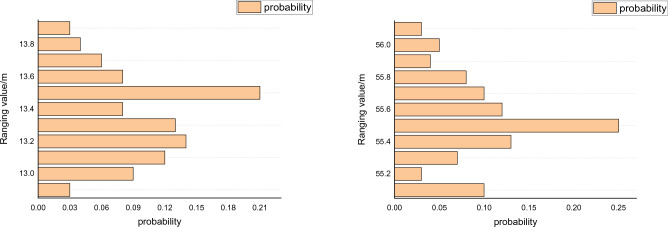
Ranging values at specific test points.

In indoor positioning applications, wireless signals can be interfered with by various factors during propagation, such as multipath effects, signal attenuation, and damping, resulting in complex time-varying characteristics of RSSI values at the exact location. Traditional fingerprint positioning algorithms typically use RSSI values as the feature vector of fingerprint signals. However, due to the instability of RSSI values, the model is prone to overfitting during offline training, leading to poor generalization performance and significant prediction errors in real-time positioning. Therefore, this method proposes to use both RSSI and TOF ranging values to form an exceptional fingerprint value. Figure 4 shows 200 measurements of ranging values obtained at several test points.

Table 1 summarizes the standard deviations of RSSI and RANGE values (without filtering) obtained at specific testing points in an indoor environment.

**Table 1:** The standard deviation of RSSI and TOF ranging values at each test point.

Test points	Number of test data	RSSI value (standard deviation)	RANGE value (standard value)/m
a	186	2.70	0.74
b	166	3.41	1.48
c	192	3.03	1.24
d	158	3.97	3.34
e	183	4.02	2.03
f	176	2.32	0.76
g	197	4.23	2.62
h	189	3.20	1.30
i	193	4.09	1.96
j	175	3.47	1.84
k	169	3.48	1.39

Results from multiple field tests indicate that the standard deviation of positioning error is more minor when using TOF range values for indoor positioning than RSSI values. Assuming that the distributions of RSSI and RANGE values follow a Gaussian-like distribution, we can incorporate the RANGE value into the fingerprint feature vector to compensate for the instability of using a single RSSI value as the fingerprint feature vector. This method can improve the accuracy and stability of positioning. Considering that the measurement error of RSSI values is more significant than that of RANGE values, a weighted scheme is adopted in this method to increase the positioning accuracy of the fusion fingerprint using both RSSI and RANGE values.

Assuming the RANGE value error follows an average distribution N(${\updelta }_{\mathrm{i}},{\upsigma }_{\mathrm{T}}^{2}$), and the RSSI distance measurement error follows a normal distribution N(${\updelta }_{\mathrm{R}},{\upsigma }_{\mathrm{R}}^{2}$), the actual distance from the unknown node A to the gateway node Bi can be calculated using the distance formula.

6 $$ \widetilde{{{\text{d}}_{{\text{i}}} }} = \sqrt {\left( {{\text{x}} - {\text{x}}_{{\text{i}}} } \right)^{2} + \left( {{\text{y}} - {\text{y}}_{{\text{i}}} } \right)^{2} } $$

Since the measured distances from the unknown node to the gateway nodes Bi are di, i = 1, 2, 3, 4, the measurement error between the measured and actual distances can be represented as ${\upvarepsilon }_{{\text{i}}} = \widetilde{{{\text{d}}_{{\text{i}}} }} - {\text{d}}_{{\text{i}}}$.

7 $$ {\upvarepsilon }_{{\text{i}}} = \widetilde{{{\text{d}}_{{\text{i}}} }} - {\text{d}}_{{\text{i}}} = \sqrt {\left( {{\text{x}} - {\text{x}}_{{\text{i}}} } \right)^{2} + \left( {{\text{y}} - {\text{y}}_{{\text{i}}} } \right)^{2} } - {\text{d}}_{{\text{i}}} $$

Therefore, the objective function for optimization can be formulated by considering the weighted sum of the errors, where the weighting factors for the RANGE and RSSI values are α and β, respectively. The weighted error sum of squares is given by:

8 $$ {\tilde{\text{e}}}\left( {{\text{x}},{\text{y}}} \right) = \sum\nolimits_{{{\text{i}} = 1}}^{4} {\upalpha \upvarepsilon _{{\text{i}}}^{2} } + \sum\nolimits_{{{\text{i}} = 1}}^{4} {\upbeta \upvarepsilon _{{\text{i}}}^{2} } = \sum\nolimits_{{{\text{i}} = 1}}^{4} {\upalpha \left( {\widetilde{{{\text{d}}_{{\text{i}}} }} - {\text{d}}_{{\text{i}}} } \right)^{2} } + \sum\nolimits_{{{\text{i}} = 1}}^{4} {\upbeta \left( {\widetilde{{{\text{d}}_{{\text{i}}} }} - {\text{d}}_{{\text{i}}} } \right)}^{2} $$

Hence, the problem of finding the approximate coordinates of the unknown node A can be transformed into a nonlinear optimization problem.

9 $$ \mathop {\min }\limits_{{\left( {{\text{x}},{\text{y}}} \right) \in {\text{R}}^{2} }} \,{\tilde{\text{e}}}\left( {{\text{x}},{\text{y}}} \right) $$

**Figure 5:**
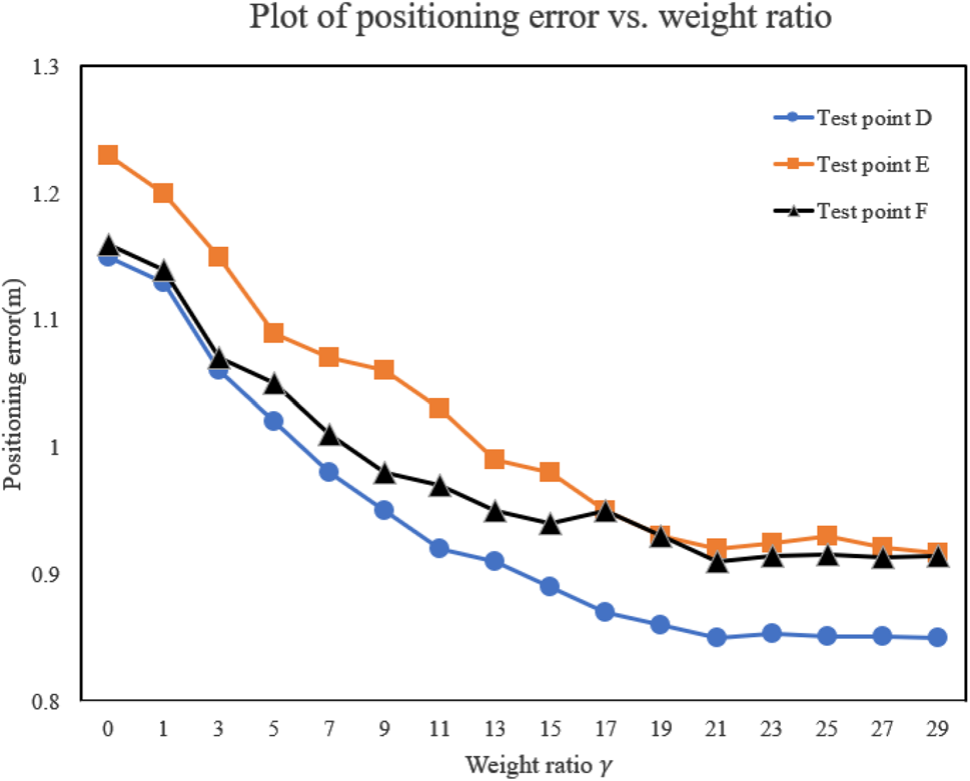
Positioning error vs weight ratio plot.

The weight ratio ${\upgamma } = {{\upalpha } \mathord{\left/ {\vphantom {{\upalpha } {\upbeta }}} \right. \kern-0pt} {\upbeta }}$ in the objective function represents the relative importance of the two distance measurement errors. Three test points were selected, and the experimental results are shown in Fig. 5. When γ = 1, i.e., when the RANGE and RSSI distance measurement errors are given equal weights, the positioning error of the unknown node is the largest. As the weight ratio γ increases, the positioning error decreases, giving higher weight to the more accurate RANGE values. However, since both distance measurement techniques have errors, it is impossible to eliminate the positioning error. When the weight ratio γ increases from 1 to 15, the improvement in positioning error is significant; when the weight ratio γ increases from 15 to 29, the improvement in positioning error is slight. Once the weight ratio γ reaches a specific value, the positioning error will stabilize. In this method, γ = 21 is selected. Equation (10) represents a weighted formula.

10 $$ {\text{RSSI}} - {\text{RANG}}_{{\text{i}}} = \frac{{{\upalpha } \times {\text{RANG}}_{{\text{i}}} + {\upbeta } \times {\text{RSSI}}_{{\text{i}}} }}{{{\upalpha } + {\upbeta }}} $$

In the given equation, ${\mathrm{RSSI}-\mathrm{RANG}}_{\mathrm{i}}$ represents the weighted value formed, where α and β are the weightings for the RANGE value and the RSSI value, respectively. ${\mathrm{RANG}}_{\mathrm{i}}$ and ${\mathrm{RSSI}}_{\mathrm{i}}$ denote the RANGE value and the RSSI value, respectively.

In indoor localization scenarios, measurement results are affected by random additive Gaussian noise and environmental changes, which may cause measurement values to deviate from the standard bell curve. Nevertheless, the actual measurement values still exhibit the characteristics of Gaussian normal distribution. Due to environmental changes during the measurement process, some values may deviate far from the mean and be affected by gross errors. Therefore, in order to eliminate the influence of these measurements on the performance of the fingerprint localization model, this method adopts the Gaussian filtering method to filter the measurement values, filtering out the measurement values that deviate far from the mean, thereby improving the accuracy and robustness of the fingerprinting localization model.

For each reference point, the mean value ${\upmu }_{\mathrm{l}}$ (mean value of each column) and standard deviation ${\upsigma }_{\mathrm{l}}$ (standard deviation of each column) of the fingerprint matrix R_i_ collected is calculated for each dimension $\mathrm{l}$ = 1, 2, …, 2M. Assuming that each column of the training set data collected follows a normal distribution, its probability density function is denoted by f(x). Thus, f(x) can be expressed as:

11 $$ {\text{f}}\left( {\text{x}} \right) = \frac{1}{{\sqrt {2\uppi \upsigma _{{\text{l}}} } }} \times {\text{e}}^{{\left( { - \frac{{\left( {{\text{x}} -\upmu _{{\text{l}}} } \right)^{2} }}{{2\upsigma _{{\text{l}}}^{2} }}} \right)}} $$

The mean μ of a normal distribution describes the central location of the distribution. Gaussian filtering can leverage the normal distribution’s characteristics to improve signal quality and construct more stable and effective fingerprint databases. The Gaussian filter eliminates noise by averaging the pixel values around each pixel, and the weight used in calculating the weighted average is a Gaussian function. This method smooths out signal values far from the mean, thereby improving the stability and reliability of the signal.

A threshold probability θ is set to represent the probability that signal values are distributed in the interval $\left[{\upmu }_{\mathrm{l}}-{\uplambda }_{\mathrm{l}},{\upmu }_{\mathrm{l}}+{\uplambda }_{\mathrm{l}}\right]$, where ${\uplambda }_{\mathrm{l}}$ can be determined using the training set data and θ. Specifically, according to Eq. (11), assuming that each column of data in the training set follows a normal distribution with probability density function f (x), ${\uplambda }_{\mathrm{l}}$ satisfies:

12 $$ \int_{{{\upmu }_{{\text{l}}} - {\uplambda }_{{\text{l}}} }}^{{{\upmu }_{{\text{l}}} + {\uplambda }_{{\text{l}}} }} {{\text{f}}\left( {\text{x}} \right){\text{dx}} = {\uptheta }} $$

After obtaining ${\uplambda }_{\mathrm{l}}$ from Eq. (12), the reasonable range of each element in column l of R_i_ is $\left[{\upmu }_{\mathrm{l}}-{\uplambda }_{\mathrm{l}},{\upmu }_{\mathrm{l}}+{\uplambda }_{\mathrm{l}}\right]$. Signal values within this range are considered reliable, while those outside are unreliable. Finally, for each element ${\mathrm{R}}_{\mathrm{i}.\mathrm{l}}^{\mathrm{k}}$ in R_i_, if ${\mathrm{R}}_{\mathrm{i}.\mathrm{l}}^{\mathrm{k}}$∈$\left[{\upmu }_{\mathrm{l}}-{\uplambda }_{\mathrm{l}},{\upmu }_{\mathrm{l}}+{\uplambda }_{\mathrm{l}}\right]$, it is retained; otherwise, ${\mathrm{R}}_{\mathrm{i}.\mathrm{l}}^{\mathrm{k}}={\upmu }_{\mathrm{l}}$.

**Figure 6:**
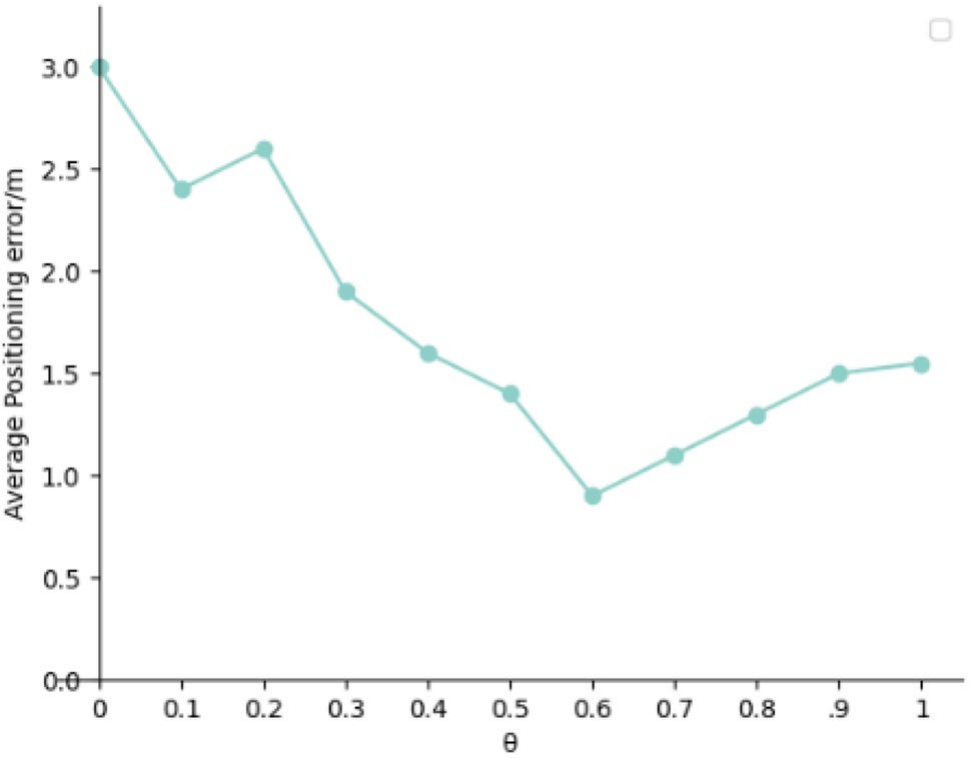
The average positioning error varies with threshold probability θ.

As**θ** approaches 1, and the original signal values are not filtered, leading to more significant localization errors. However, when θ approaches 0, the filtering of the samples is too strict, which may result in the loss of many valid signal values and the loss of the characteristics of the fingerprint point. In this method, θ is set to 0.6. Figure 6 shows the variation of the average localization error of this algorithm with the threshold probability θ.

**Figure 7:**
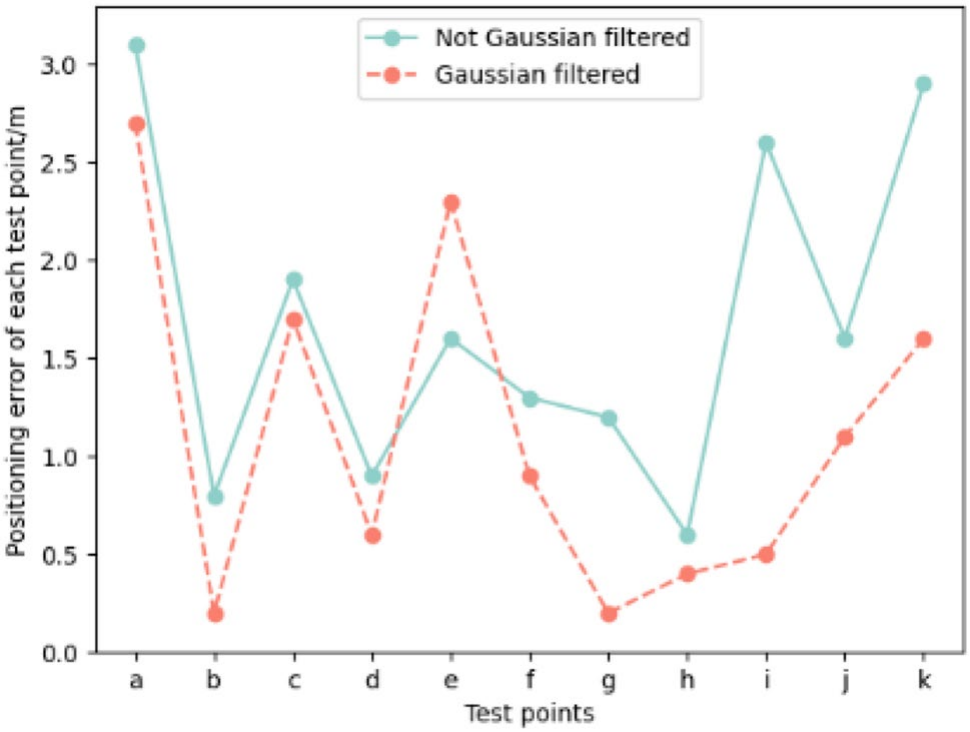
Gaussian filter processing data comparison chart.

During the data collection process, sampling points were randomly selected and data was collected over varying time periods. This approach aimed to ensure a more comprehensive and realistic representation of the collected data. Figure 7 shows that during the offline fingerprint acquisition phase, some test point positioning errors are affected by Gaussian filtering. The solid line represents the localization accuracy obtained by directly storing the fingerprint data in the database without Gaussian filtering. In contrast, the dashed line represents the localization accuracy obtained after applying Gaussian filtering and storing the data in the database. The figure shows that, except for one test point, the localization accuracy after Gaussian filtering is generally better than that without Gaussian filtering for most of the 10 test points. This indicates that using Gaussian filtering for preprocessing the signal values in each dimension during the offline fingerprint collection phase can significantly improve localization accuracy.

**Figure 8:**
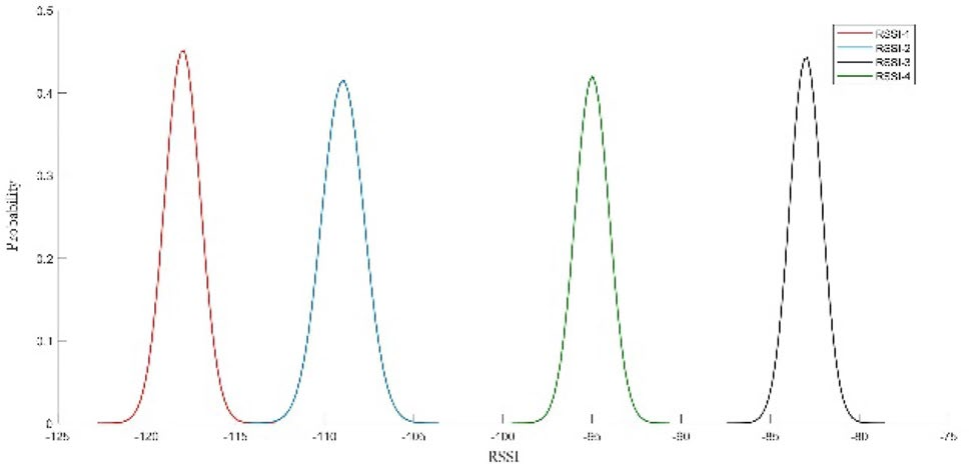
The RSSI value of test point e.

Figure 8 shows the RSSI values between test point e and four gateways after Gaussian filtering.

### Storing in the fingerprint database

Using the preprocessed fingerprint matrix R_i_ of each reference point and the location coordinates ${\mathrm{P}}_{\mathrm{i}}=\left({\mathrm{x}}_{\mathrm{i}} ,{\mathrm{y}}_{\mathrm{i}}\right)$, a fingerprint database D is established, where R_i_∈${\mathrm{R}}^{2\mathrm{m}}$ and serves as the training set for the RF algorithm.

In experiments 2 and 3, obtaining the RSSI-RANGE values of the entire deployment area was not feasible. Measuring the RSSI-RANGE values throughout the entire deployment area was costly and unnecessary. This method utilizes the Kriging method for interpolation. Kriging is a statistical method used for spatial interpolation, commonly employed to estimate attribute values at unknown locations. The core idea of Kriging is to estimate the values at unknown points based on the spatial correlation among known points. In this article, the known points are the RSSI-RANGE values obtained within the deployment area. This method utilizes a semivariogram function to describe the variation in signal strength. The semivariogram function quantifies the spatial correlation between two points, representing the degree of signal strength variation. Based on the distances between known points and the mathematical model of the semivariogram function, the value at an unknown point can be estimated. Equation (13) represents the Kriging formula:

13 $$\mathrm{Z}\left({\mathrm{s}}_{0}\right)=\sum_{\mathrm{i}=1}^{\mathrm{N}}{\uplambda }_{\mathrm{i}}\mathrm{Z}\left({\mathrm{s}}_{\mathrm{i}}\right)$$

where $\mathrm{Z}\left({\mathrm{s}}_{\mathrm{i}}\right)$ represents the measured value at the i-th location, ${\uplambda }_{\mathrm{i}}$ denotes the Kriging weight associated with the measurement at the i-th location, ${\mathrm{s}}_{0}$ represents the prediction location, and N is the number of measured values.

The calculation of Kriging weights relies on the model of the semivariogram function. Due to the influence of buildings, walls, obstacles, and other factors on LoRa signal propagation, the propagation model typically exhibits nonlinear attenuation and multipath effects. Therefore, in this research, the selected semivariogram function model is the Gaussian model.

14 $$\mathrm{y}\left(\mathrm{h}\right)={\mathrm{C}}_{0}+{\mathrm{C}}_{1}\times \left(1-{\mathrm{e}}^{\left(-\frac{{\mathrm{h}}^{2}}{{\mathrm{r}}^{2}}\right)}\right)$$

where $\mathrm{y}\left(\mathrm{h}\right)$ represents the value of the semivariogram function, h denotes the distance between known points, and ${\mathrm{C}}_{0}$, ${\mathrm{C}}_{1}$, and r are parameters of the model. ${\mathrm{C}}_{0}$ represents the baseline value, ${\mathrm{C}}_{1}$ represents the magnitude of signal strength variation, and r represents the scale parameter of correlation.

This method utilizes the Kriging library in Python to train the Kriging model using a fingerprint database. The centre coordinates of the grids without fingerprint points were selected as the interpolation coordinates. Table 2 shows some of the generated RSSI interpolations in Experiment 2.

**Table 2:** Partial RSSI interpolation data.

Coordinate	RSSI-1	RSSI-2	RSSI-3	RSSI-4
(4.5,1.75)	− 67	− 91	− 111	− 126
(13.5,1.25)	− 68	− 78	− 96	− 105
(64.5,1.75)	− 99	− 101	− 88	− 55
(34.5,0.75)	− 74	− 63	− 73	− 83
(25.5,0.25)	− 72	− 64	− 92	− 103

### Construction of offline model

Before constructing the offline model, the obtained data were processed using the PSO-RF algorithm to obtain the optimal fingerprint values.

**Figure 9:**
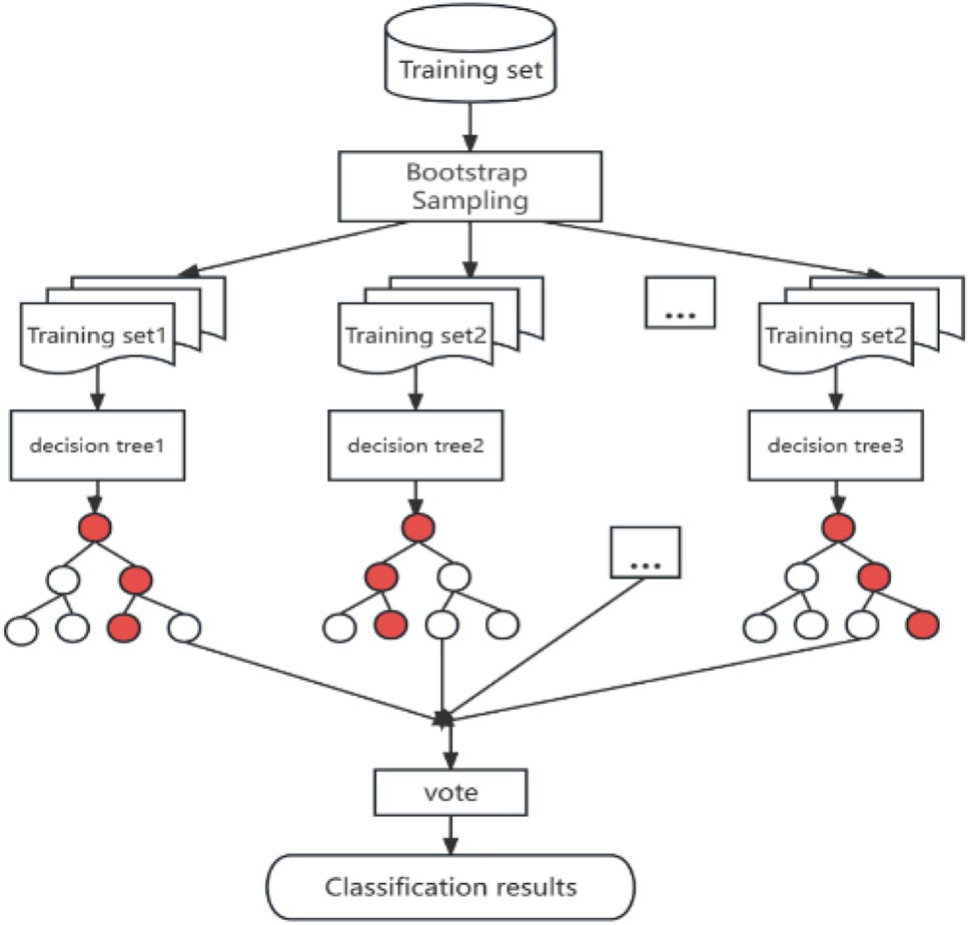
RF model.

Random Forest (RF) algorithm^26^ is a very flexible method that can automatically handle missing data, nonlinear relationships, and combinations of multiple features. The RF model is shown in Fig. 9.

The number of trees and the number of feature selections in the RF algorithm have an essential impact on the performance and complexity of the model. However, they usually require manual adjustment, a tedious task requiring a lot of trial and error. By using the PSO algorithm to optimize the hyperparameters of the RF algorithm, the performance and generalization ability of the model can be improved while reducing the time and energy cost of manual tuning.

Particle Swarm Optimization (PSO) is an optimization algorithm based on swarm intelligence^27^. This algorithm considers the optimisation problem to find the optimal global solution in a multidimensional space, and each solution is considered a particle in space.

These particles search for the optimal solution by continuously adjusting their position and velocity. In the search process, Eqs. (15) and (16) are used to update the position and velocity of each particle. In order to improve the convergence speed of the algorithm, a weight factor w is introduced. The particle representation is called velocity, but the distance and direction the particle will move in the next iteration is a position vector.

15 $${\mathrm{v}}_{\mathrm{id}}^{\mathrm{k}+1}=\upomega {\mathrm{v}}_{\mathrm{id}}^{\mathrm{k}}+{\mathrm{c}}_{1}{\mathrm{r}}_{1}\left({\mathrm{p}}_{\mathrm{id},\mathrm{pbest}}^{\mathrm{k}}-{\mathrm{x}}_{\mathrm{id}}^{\mathrm{k}}\right)+{\mathrm{c}}_{2}{\mathrm{r}}_{2}\left({\mathrm{p}}_{\mathrm{id},\mathrm{gbest}}^{\mathrm{k}}-{\mathrm{x}}_{\mathrm{id}}^{\mathrm{k}}\right)$$

16 $${\mathrm{x}}_{\mathrm{id}}^{\mathrm{k}+1}={\mathrm{x}}_{\mathrm{id}}^{\mathrm{k}}+{\mathrm{v}}_{\mathrm{id}}^{\mathrm{k}+1}$$

In the equation, k represents the number of iterations, $\upomega$ represents the inertia weight, ${\mathrm{c}}_{1}$ represents the individual learning factor, ${\mathrm{c}}_{2}$ represents the group learning factor, and ${\mathrm{r}}_{1}{\mathrm{r}}_{2}$ is random numbers in the interval [0, 1] to increase the randomness of the search. ${\mathrm{v}}_{\mathrm{id}}^{\mathrm{k}}$ represents the velocity vector of particle i in the d-th dimension in the k-th iteration, ${\mathrm{x}}_{\mathrm{id}}^{\mathrm{k}}$ represents the position vector of particle i in the d-th dimension in the k-th iteration, ${\mathrm{p}}_{\mathrm{id},\mathrm{pbest}}^{\mathrm{k}}$ represents the historical best position of particle i in the dth dimension in the k-th iteration, i.e., the best solution found by the i-th particle (individual) after the k-th iteration, and ${\mathrm{p}}_{\mathrm{id},\mathrm{gbest}}^{\mathrm{k}}$ represents the historical best position of the group in the dth dimension in the k-th iteration, i.e.,the best solution found by the entire particle swarm after the k-th iteration.

The PSO-RF algorithm randomly initializes a swarm of particles for the hyperparameters of the decision tree, such as the number of trees (n_estimators) and the maximum depth (max-depth). It then calculates the corresponding fitness values and continuously updates the particles’ velocities and positions to achieve the best fitness value, thereby obtaining the optimal hyperparameters for the RF model, n_estimators and max_depth, which in turn improves the convergence speed and prediction performance of the RF model.

**Figure 10:**
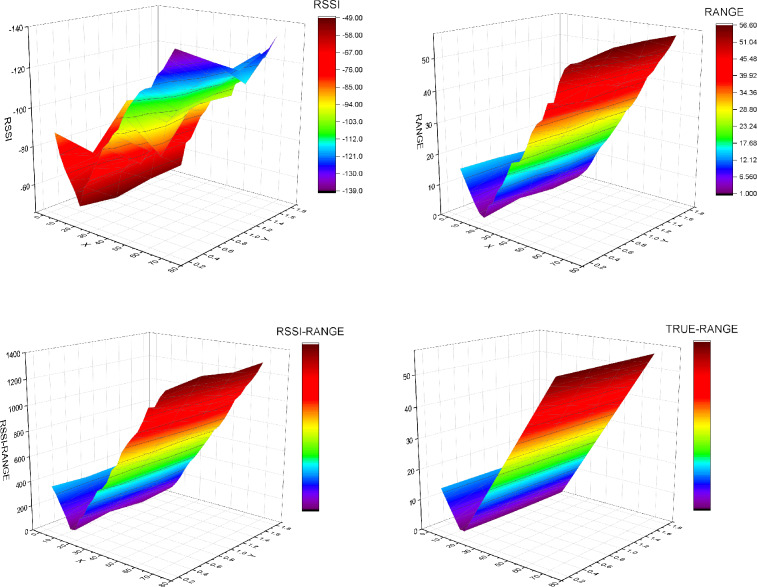
Fingerprint method comparison chart.

Figure 10 compares the single RSSI fingerprint, single RANGE fingerprint, RSSI-RANGE fingerprint, and actual distance value of Gateway 1 collected in Experiment 2 after being processed by the algorithm.

### Online real-time positioning phase

During actual positioning, LoRa devices are used to collect real-time RSSI-RANGE features at a specific location for t times. The data is preprocessed using a median average filtering method. The median averaging filter is a signal processing technique used to reduce noise in a signal. It smooths the signal by calculating the median value of the data points within a window. The main advantage of the median averaging filter is its effectiveness in reducing noise, especially in the presence of outliers or impulse noise. Compared to other averaging filter methods, the median averaging filter performs better in preserving signal edges and details because it is not affected by outliers. The maximum and minimum values of the t RSSI-RANGE features at the exact location are removed, and the remaining data is averaged to obtain a unique fingerprint feature. This fingerprint feature is then input into the offline training model obtained in section “Construction of offline model” to calculate the coordinates of the target node.

At a particular location for t times, LoRa devices continuously collect fingerprint feature vectors, resulting in t records denoted as:

$$ {\text{W}} = \left[ {\begin{array}{*{20}l} {{\text{R}}_{1}^{1} { }} \hfill & {\quad {\text{R}}_{2}^{1} } \hfill & {\quad \cdots } \hfill & {\quad {\text{R}}_{{\text{l}}}^{1} } \hfill & {\quad \cdots } \hfill & {\quad {\text{R}}_{{2{\text{n}}}}^{1} } \hfill \\ {{\text{R}}_{1}^{2} { }} \hfill & {\quad {\text{R}}_{2}^{2} } \hfill & {\quad \cdots } \hfill & {\quad {\text{R}}_{{\text{l}}}^{2} } \hfill & {\quad \cdots } \hfill & {\quad {\text{R}}_{{2{\text{n}}}}^{2} } \hfill \\ \vdots \hfill & {\quad \vdots } \hfill & {} \hfill & {\quad \vdots } \hfill & {} \hfill & {\quad \vdots } \hfill \\ {{\text{R}}_{1}^{t} { }} \hfill & {\quad {\text{R}}_{2}^{t} } \hfill & {\quad \cdots } \hfill & {\quad {\text{R}}_{{\text{l}}}^{t} } \hfill & {\quad \cdots } \hfill & {\quad {\text{R}}_{{2{\text{n}}}}^{t} } \hfill \\ \end{array} } \right] $$

For ${\mathrm{R}}_{\mathrm{l}}^{\mathrm{t}}$, when l = 2j-1 (odd columns), it represents the RSSI fingerprint value from the j-th gateway device obtained in the tth collection; when l = 2j (even columns), it represents the range fingerprint value obtained based on the ranging engine mode from the j-th gateway device in the tth collection. Here, l = 1, 2, …, 8, and j = 1, 2, …, 4. t = 1, 2, …, 10. For each column, the maximum and minimum values are removed, and the average value of the remaining data is denoted as ${\mathrm{R}}_{\mathrm{l}}^{\mathrm{^{\prime}}}$. The average value vector of W should be: [${\mathrm{R}}_{1}^{\mathrm{^{\prime}}},{\mathrm{ R}}_{2}^{\mathrm{^{\prime}}},\cdots ,{\mathrm{ R}}_{\mathrm{l}}^{\mathrm{^{\prime}}},\cdots ,{\mathrm{ R}}_{2\mathrm{n}}^{\mathrm{^{\prime}}}$].

### Enter the offline model

The obtained average vector of W is then used as input to the offline training model constructed in section “Construction of offline model”. The model then calculates the two-dimensional coordinates (x, y) of the location to be determined, which are taken as the final position coordinates. In this way, the actual position of the test point can be estimated.

## Experiment and results

### Experimental hardware

The experiment used 4 LoRa gateways and 1 terminal node to verify the PSO-RF-FPL algorithm. The gateways and terminal node integrated Arduino UNO R3, and the LoRa RF module used the SX1280 chip. The experiment environments were a rectangular indoor environment, a narrow indoor corridor, and a cross-room indoor environment. In the three environments, the following six situations were compared:

1. Using PSO-RF-FPL, fingerprint feature selection: RANGE value.
2. Using PSO-RF-FPL, fingerprint feature selection: RSSI value.
3. Using PSO-RF-FPL, fingerprint feature selection: RSSI-RANGE value.
4. Using FPL, fingerprint feature selection: RSSI-RANGE value.
5. Using FPL, fingerprint feature selection: RSSI value.
6. Using RF-FPL, fingerprint feature selection: RSSI-RANGE value.

The error of each algorithm was calculated, and the Euclidean distance between the actual coordinate and the estimated coordinate was used as the evaluation criterion for the positioning error:

17 $$\mathrm{Error}=\sqrt{{\left({\mathrm{x}}_{1}-{\mathrm{x}}_{2}\right)}^{2}+{\left({\mathrm{y}}_{1}-{\mathrm{y}}_{2}\right)}^{2}}$$

where (x_1_, y_1_) is the actual coordinate, and (x_2_, y_2_)is the coordinate calculated by the three algorithms.

### Rectangular indoor environment

**Figure 11:**
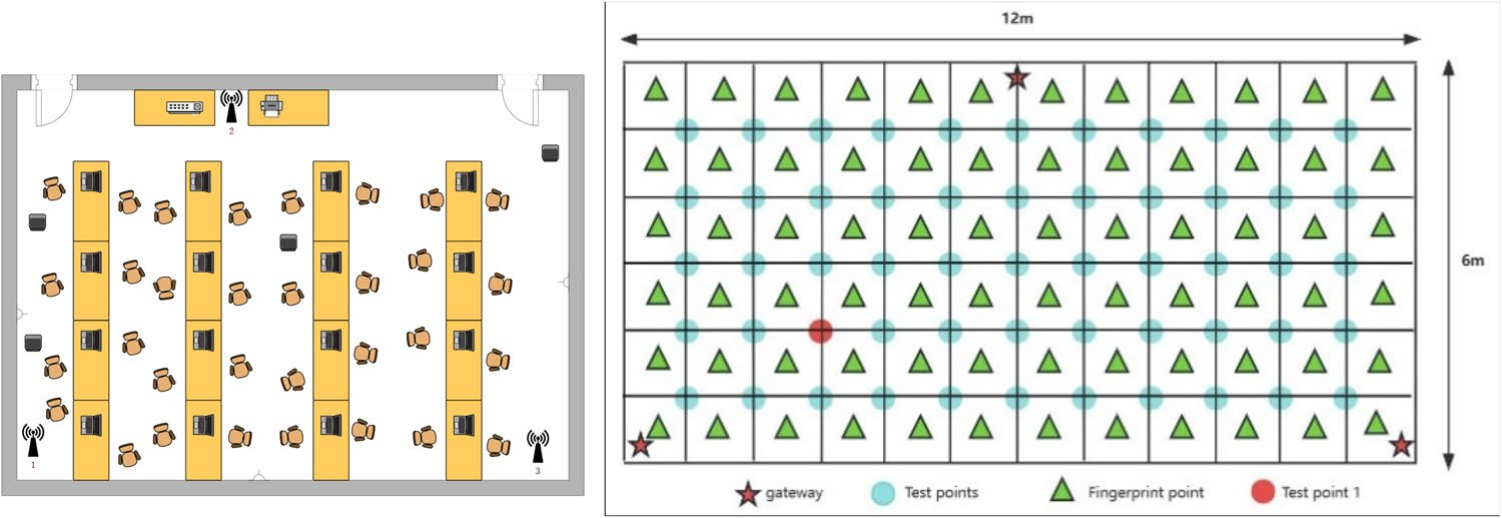
Rectangular interior floor plan.

The experimental environment is shown in Fig. 11, with a length of 12 m and a width of 6 m. A total of 55 fingerprint points and 40 test points were selected in the entire experimental area. Three gateways are deployed from left to right as 1, 2, and 3. The experimental site is divided into small grids, and a fixed LoRa gateway is deployed in each grid at positions (0, 0), (6, 6), and (12, 0), respectively.

During the offline fingerprint acquisition stage, LoRa signal features were uniformly collected 200 times as one set of fingerprint data in the grid containing fingerprint points, with an interval of 2 s between each collection, and filtered using a Gaussian filter. Each set of fingerprint data consists of two parts: the RSSI value collected by LoRa, and the RANGE value based on LoRa ranging engine mode.

The central position coordinates of the i-th grid point area are denoted as P_i_ = (x_i_, y_i_), and the fingerprint matrix R_i_ collected in the i-th grid point area is denoted as follows:

$$ {\text{R}}_{{\text{i}}} = \left( {\begin{array}{*{20}l} {{\text{R}}_{{{\text{i}}.1}}^{1} } \hfill & {{\text{ R}}_{{{\text{i}}.2}}^{1} } \hfill & {{ } \cdots } \hfill & {{\text{ R}}_{{{\text{i}}.{\text{l}}}}^{1} } \hfill & \cdots \hfill & {{\text{ R}}_{{{\text{i}}.6}}^{1} } \hfill \\ {{\text{R}}_{{{\text{i}}.1}}^{2} } \hfill & {{\text{ R}}_{{{\text{i}}.2}}^{2} } \hfill & \cdots \hfill & {{\text{ R}}_{{{\text{i}}.{\text{l}}}}^{2} } \hfill & \cdots \hfill & {{\text{ R}}_{{{\text{i}}.6}}^{2} } \hfill \\ \vdots \hfill & \vdots \hfill & \vdots \hfill & \vdots \hfill & \vdots \hfill & \vdots \hfill \\ {{\text{R}}_{{{\text{i}}.1}}^{{\text{k}}} } \hfill & {{\text{ R}}_{{{\text{i}}.2}}^{{\text{k}}} } \hfill & \cdots \hfill & {{\text{ R}}_{{{\text{i}}.{\text{l}}}}^{{\text{k}}} } \hfill & \cdots \hfill & {{\text{ R}}_{{{\text{i}}.6}}^{{\text{k}}} } \hfill \\ \vdots \hfill & \vdots \hfill & \vdots \hfill & \vdots \hfill & \vdots \hfill & \vdots \hfill \\ {{\text{R}}_{{{\text{i}}.1}}^{100} } \hfill & {{\text{ R}}_{{{\text{i}}.2}}^{100} } \hfill & \cdots \hfill & {{\text{ R}}_{{{\text{i}}.{\text{l}}}}^{100} } \hfill & \cdots \hfill & {{\text{ R}}_{{{\text{i}}.6}}^{100} } \hfill \\ \end{array} } \right) $$

For ${\mathrm{R}}_{\mathrm{i}.\mathrm{l}}^{\mathrm{k}}$, when l = 2j − 1 (odd column), it Indicates the RSSI fingerprint value of the received signal strength obtained from the j-th gateway device based on the communication mode acquired for the k-th time at the i-th fingerprint grid point area. When l = 2j (even column), it represents the RANGE value obtained from the j-th anchor device based on the ranging engine mode in the k-th collection at the i-th fingerprint grid point region, where i = 1, 2, …, 55, j = 1, 2, 3, l = 1, 2, …, 6, and k = 1, 2, …, 200.

**Figure 12:**
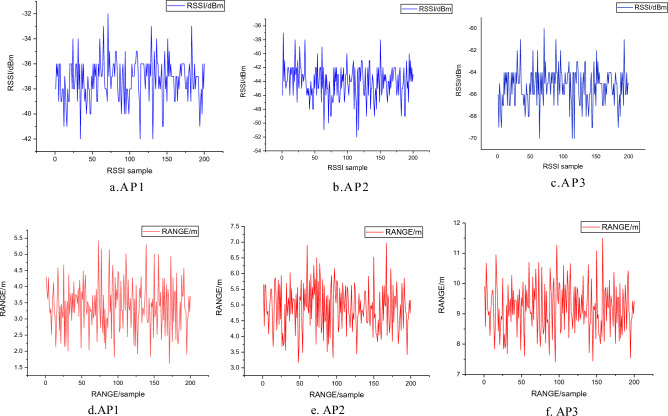
The gateway collects the RSSI value and RANGE value of test point 1.

As shown in Fig. 12a–c represent the RSSI values collected by three gateways at test point 1, while d, e, and f represent the RANGE values collected by three gateways at test point 1.

### Narrow and elongated indoor corridor environment

**Figure 13:**
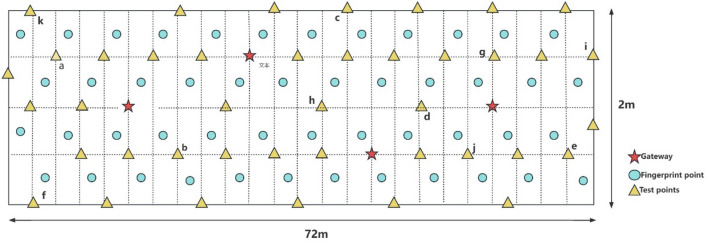
Corridor diagram.

The experimental environment for the narrow indoor corridor is shown in Fig. 13, which is 72 m long and 2 m wide, with an area of approximately 144 m^2^. A total of 48 fingerprint points and 41 test points were selected in the entire experimental area. Four gateways were deployed from left to right, numbered 1 to 4.

Similar to the previous experiment, LoRa gateways were deployed at fixed positions (15, 1), (30, 1.5), (45, 0.5), and (60, 1), and the remaining grids were interpolated using the Kriging model. The fingerprint collection method was the same as described in section “Rectangular indoor environment”.

### Cross-room indoor environment

**Figure 14:**
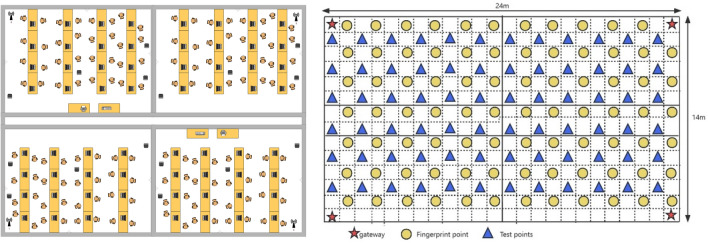
Cross-room interior diagram.

The experimental environment and floor plan of the indoor environment with multiple rooms is shown in Fig. 14, with a length of 24 m and a width of 14 m. A total of 83 fingerprint points and 72 test points were selected in the entire experimental area. Four gateways were deployed, with Gateway One at the bottom left, Gateway Two at the bottom right, Gateway Three at the top right, and Gateway Four at the top left.

Similarly, LoRa gateways were deployed in fixed positions at (0, 0), (24, 0), (24, 14), and (0, 14) in these grids. Fingerprint collection was performed using the same method described in section “Rectangular indoor environment”, and Kriging interpolation was used for the remaining grids.

### Experimental results

The experimental results for the three scenarios are shown in the Table 3 below.

**Table 3:** Average positioning error for six cases.

Experiment	Algorithm	Fingerprint features	Average positioning error/m	Standard deviation of positioning error/m	Sample standard deviation/m
Experiment 1	PSO-RF-FPL PSO-RF-FPL PSO-RF-FPL	RANGE	0.97	1.23	1.27
		RSSI	1.96	2.58	1.31
		RSSI-RANGE	0.85	1.09	1.28
	FPL FPL	RSSI-RANGE	1.24	1.76	1.41
		RSSI	2.01	2.79	1.39
	RF-FPL	RSSI-RANGE	1.08	1.72	1.59
Experiment 2	PSO-RF-FPL	RANGE	0.94	1.12	1.19
	PSO-RF-FPL	RSSI	1.87	2.47	1.32
	PSO-RF-FPL	RSSI-RANGE	0.82	0.99	1.21
	FPL	RSSI-RANGE	1.17	1.80	1.54
	FPL	RANGE	1.98	2.84	1.43
	RF-FPL	RSSI-RANGE	1.03	1.59	1.54
Experiment 3	PSO-RF-FPL	RANGE	1.12	1.46	1.30
	PSO-RF-FPL	RSSI	2.13	2.97	1.39
	PSO-RF-FPL	RSSI-RANGE	0.98	1.34	1.37
	FPL	RSSI-RANGE	1.49	2.13	1.43
	FPL	RSSI	2.67	3.16	1.18
	RF-FPL	RSSI-RANGE	1.35	1.96	1.45

**Figure 15:**
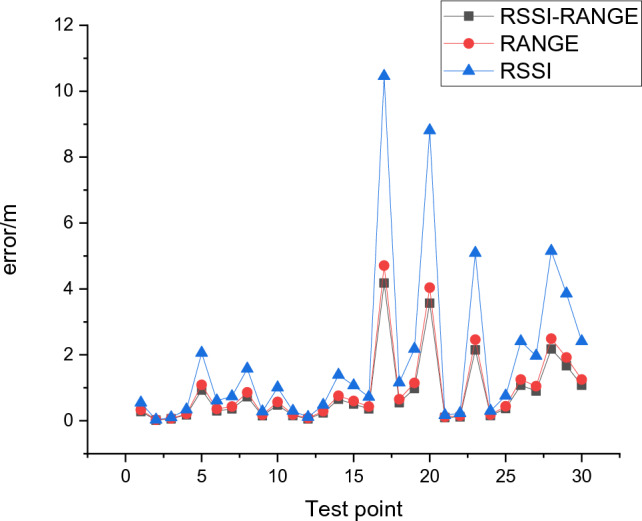
Error comparison of different fingerprint values.

Based on experiment 2, it can be observed that in experiment (1), (2), and (3) comparisons, the third case, which uses both RSSI and RANGE values for fingerprinting, has an average positioning error of 0.82 m, the SSD is 1.21 m. In the second case, where only RSSI values are used, the average positioning error is 1.87 m, the SSD is 1.32 m. while in the first case, where only RANGE values are used, the average positioning error is 0.94 m, the SSD is 1.19 m. Figure 15 shows the error comparison of 30 testing points using different fingerprinting values.

**Figure 16:**
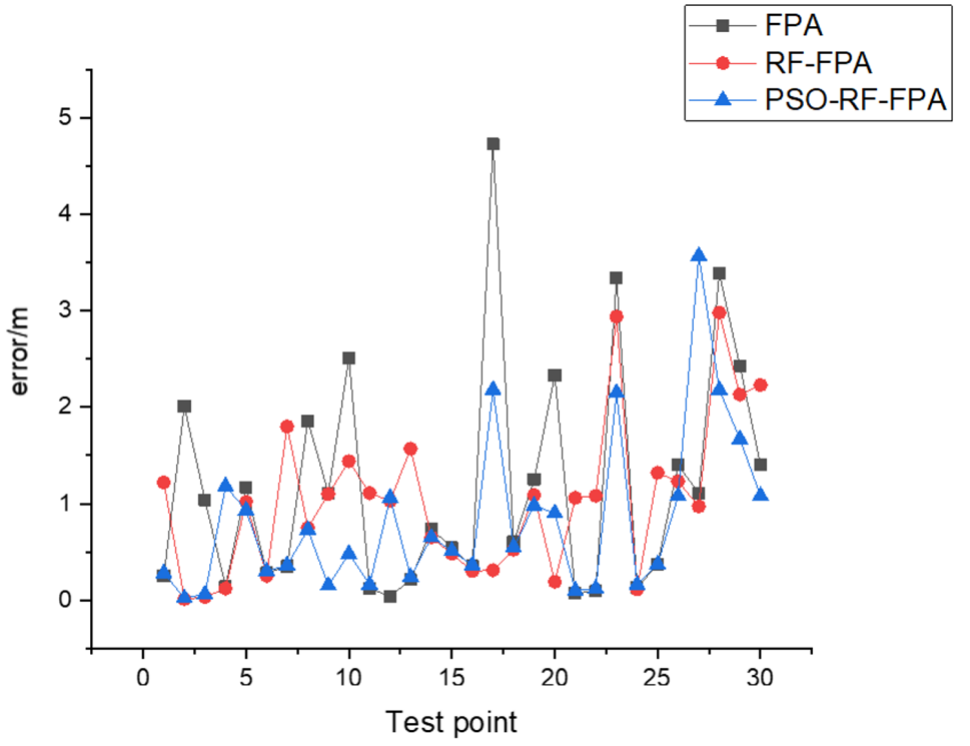
Comparison of different algorithm errors.

In experiments (3), (4), and (6) comparisons, without using the RF algorithm to optimize the data, the average positioning error is 1.17 m, the SSD is 1.54 m. After processing the data using the RF algorithm optimized by the PSO algorithm, the average positioning error is the lowest at 0.82 m, the SSD is 1.21 m. Figure 16 shows the error comparison of 30 testing points using different fingerprinting values.

**Figure 17:**
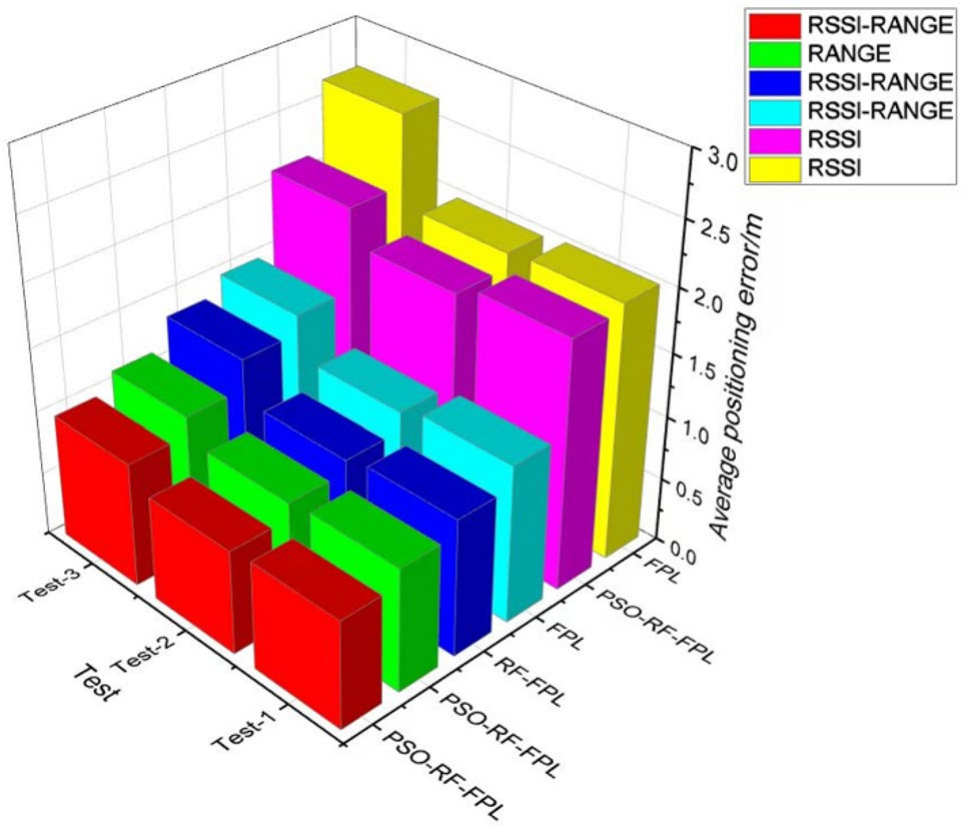
Comparison of the overall error of the experiment.

It can be seen by comparing experiments 1, 2, and 3. It can be seen that the presence of many electronic devices and obstacles, such as walls in the indoor environment, leads to a slight decrease in positioning accuracy. However, the proposed method in this method is still effective in improving positioning accuracy. Figure 17 shows the overall error comparison of experiments 1, 2, and 3.

## Conclusion

In this method, we address the weakness of traditional Fingerprint-based Positioning Systems (FPL), which is the susceptibility of the Received Signal Strength Indicator (RSSI) to interference that leads to poor model quality during offline training. We propose two improvements: (1) the addition of Time of Flight (TOF) ranging fingerprint to improve fingerprint stability by combining new weighted fingerprint values, and (2) Gaussian and median filtering for fingerprint preprocessing during fingerprint database creation online positioning, respectively. We eliminate gross fingerprint errors and use the Kriging method for interpolation, followed by the PSO-RF-FPL algorithm to build an optimal offline fingerprint database. We demonstrate the effectiveness and reliability of our proposed approach through on-site indoor experiments.

The results show that adding TOF ranging values in the three experimental scenarios increased the positioning accuracy by 53–57% while using the PSO-RF-FPL algorithm improved the positioning accuracy by 25–31%. When both improvements were combined, the positioning accuracy increased by 58–63%, indicating the effectiveness of our proposed algorithm.

This study employs various gateway placement strategies in practical experiments. In different scenarios, the aim is to use as few gateways as possible based on the size of the experimental environment. Although an increase in the number of gateways improves localization accuracy, the purpose of this paper is to validate the effectiveness of the proposed method. Therefore, determining the appropriate number and placement of gateways in real-world scenarios is one of the future research directions.

Future work will focus on.

1. Increasing the number of gateways to improve positioning accuracy.
2. Applying this method to indoor personnel positioning to identify positions as accurately as possible.
3. Using this method for multi-floor indoor positioning to improve diversity in real-life applications.

## Data Availability

The datasets generated and/or analysed during the current study are not publicly available due. The data is collected at the experimental field and is not universal but are available from the corresponding author on reasonable request.
